# Medical Opinion and Movement

**Published:** 1911-05-27

**Authors:** 


### Medical Opinion and Movement.

### Basedow's Disease and Stature.

In an article in the *Norddeutscher Medizinische Archiven*, Dr. Holmgren discusses the relation between exophthalmic goître with tachycardia and height of stature. From examination of a number of cases, he concludes that a patient suffering from Basedow's disease during the period of growth is liable to attain a stature above the average. Thus, of 33 patients under twenty-two years of age, 28 were above the average height and 5 were under. On the other hand, of 31 patients above the age of twenty-two, 15 were above the average and 16 below. No marked difference from the normal was found in patients suffering from goître without tachycardia. Radioscopic examination of the hands showed that the patients affected with goître and of a height above the normal obeyed the general law according to which ossification of the conjugal cartilage takes place earlier in tall people than in others. From Italian literature on the subject, Dr. Holmgren concludes also that exophthalmic goître predisposes to greater height in stature, for it appears that in goître districts the height is rather greater than in neighbouring districts. The author characterises the typical goître patient thus: Tall and blond, menstruation early, full neck, tachycardia and slight tremors, fair hair and brilliant eyes, nervous and lively aspect, with an intelligence often above the average.

### Abdominal Drainage.

In a contribution to the *Revue de Chirurgie*, Drs. Caraven and Basset discuss the question of ulceration of the iliac arteries from pressure by drainage-tubes. Although most surgeons are probably fully alive to this danger attending the use of abdominal drainage-tubes, and in consequence take every care to avoid proximity to the vessels in placing the tubes, the case which forms the basis of this communication is not without interest. The patient was a boy aged thirteen, with a large appendicular abscess. The abscess was opened freely and two drainage-tubes inserted. All went well till the fifth day, when a severe hæmorrhage occurred. This increased seriously on withdrawing the tubes, necessitating immediate compression of the abdominal aorta. The source of the hæmorrhage was carefully sought for, and found to be due to ulceration of the external iliac artery. A double ligature was applied above and below the perforation and the hæmorrhage arrested. The perforation was about 8 mm. in length on the anterior surface of the vessel and at the point corresponding to the lower ends of the drainage-tubes. Examination of the tubes showed that they had been bent at two places, pointing to their exercising considerable pressure on the floor of the iliac fossa. A second case is also reported of ulceration of both external iliacs subsequent to an operation on the ureters. The authors point out that there appears to be no danger in applying the ligatures to the vessel in a septic field. In order to avoid this, in such cases it would be necessary to apply the upper ligature to the common iliac and the lower at the crural canal, which would incur the risk of spreading the infection, and would seriously interfere with subsequent compensatory circulation by means of the collateral anastomoses.

### Intravenous Etherisation.

At the recent German Surgical Congress an interesting discussion was inaugurated by Dr. Kümmell on the method of etherisation by intravenous injection. He considers the method particularly well adapted for operations on the head and neck, as it entirely avoids interference with the surgeon in the field of operation. The injection of saline keeps up the strength of the patient against the loss of blood involved in the operation, and the ether acts as a direct cardiac stimulant, so that in feeble subjects the pulse is generally better after than before operation. Recovery from narcosis is rapid and without malaise and there is no vomiting. He considers the dangers of intravenous etherisation have been exaggerated. The weak point in the method is the formation of thrombi. The author has often observed local thrombi in the vein at the site of injection. They are painful, but no other symptoms have occurred, and the author points out that these also occur with simple saline injections especially if adrenalin is added. In cases in which a subsequent autopsy has taken place no changes in the kidneys or heart attributable to the etherisation have been observed. He considers, however, the method is contra-indicated in arterio-sclerosis or myocarditis. The urine and blood show no change after the narcosis. Dr. Kümmell further gave details of technique by which he was able to avoid the formation of thrombi. The patients are anæsthetised in three to fifteen minutes. Usually 200 to 300 c.c. of 5 per cent. ether solution are used. In the subsequent discussion several surgeons spoke in praise of this method of etherisation. Dr. Hagemann had observed hæmoglobinuria follow narcosis.

### Apical Phthisis.

At the recent German Medical Congress, Dr. Bacmeister of Freibourg sought to explain the cause of the frequent localisation of tuberculosis at the apices of the lung by experiments he has carried out on rabbits. The hypothesis has been put forward that this localisation of the disease at the apices is due to more or less pronounced external pressure in this region. In order to test the validity of this supposition, he produced a stenosis of the upper part of the thorax in young growing rabbits by applying a metallic ring at the level of the first rib. In this way he mechanically caused an atelectasis of the apices of the lungs. He then studied the course of infection by tubercle bacilli by inhalation and through the blood channel. Intravenous injection of an emulsion of tubercle bacilli in animals with the mechanically constricted thorax resulted in a tuberculous infection strictly limited to the apices, the rest of the lungs remaining absolutely intact. Experiments with an emulsion of cinnabar gave analogous results. The grains of cinnabar were localised to the apices. If, however, cinnabar emulsion is injected into a normal rabbit, the cinnabar grains are diffused through the lung and are only arrested by the lymphatic glands. The constriction of the thorax appears to render the pulmonary filter more dense, so as arrest the cinnabar or tubercle bacilli brought to it by the blood. In another series of experiments the animals were exposed to an atmosphere containing microbic dust. In these cases, in which infection occurred by aspiration of a large quantity of bacilli, there was no localisation of the disease to the apices. The disease was always generalised. Finally, in a further series of experiments, an inguinal gland was infected with tubercle bacilli. There resulted a tuberculous bronchitis and peri-bronchitis, almost entirely localised to the apices.

### Systematic Examination of the Stomach.

At a recent meeting of the Paris Academy of Médecine Drs. Aubourg and Lebon showed that systematic examination of the stomach by means of the *x*-rays gives precise information regarding retardation of the digestion as the result of atony of the gastric muscle. By directly exciting the fibres with a sound through which a galvanic current was passed the contractions of the stomach appeared immediately with a rapidity of succession and an intensity which allowed of quick evacuation of even the most atonic stomachs. This painless method, the effects of which are borne out by examination with the *x*-rays during the actual application of the electricity, would seem indicated in cases of gastric atony and ptosis.

### Calcined Magnesia for Laryngeal Papilloma.

The treatment of multiple laryngeal papillomata is a much discussed question. Some surgeons advocate tracheotomy solely with the idea of putting the larynx totally at rest, others advise thyrotomy, and of late laryngostomy has been tried with a certain measure of success. Claoué, in a communication made to the Society of Médecine of Bordeaux describes two cases of the disease in which none of these methods could be carried out, and in which he accordingly removed the tumours carefully by the direct route under chloroform, only to find a month later that recurrence was complete. It then struck him that the papillomata had much in common histologically with the ordinary warts of childhood, and he accordingly determined to give each of his patients a course of treatment with calcined magnesia. The first was given massive daily doses of 80 grains of the drug over a period of two weeks, after which improvement was marked. A rest of two weeks was then allowed, followed by 8 grains daily for four months, when the cure was found to be complete. The second patient was treated by regular doses of 8 grains over a period of five months, at the end of which he had recovered his voice and all trace of papillomata had disappeared.

### Researches upon Hepatic Cirrhosis.

Some most extensive and patient researches into the pathology of cirrhosis are reported by Dr. F. B. Mallory, who distinguishes five separate types of lesions which may end in sclerosis of the liver. Of these five lesions one is acute, the other four are chronic; one of the questions he has set himself to answer is whether it is possible, in the case of a given cirrhosed liver, to say by microscopical examination how that particular case has originated, and to this question he is inclined to give a positive answer in most cases, and perhaps in all. Toxic cirrhosis is the name he gives to the acute process which may follow extensive central necrosis of the liver cells. When all the liver cells of a lobule are destroyed, the bile-ducts grow out a certain distance towards the hepatic vein, but they do not produce liver cells; the latter regenerate only from liver cells, never from bile-duct epithelium. Fibroblasts do not proliferate, he says, when liver cells alone are destroyed, but only when they themselves have been injured or irritated. So-called alcoholic cirrhosis is characterised by a peculiar hyaline degeneration preceding the necrosis of the cells. Here contraction of the connective tissue frequently compresses groups of liver cells so that they may resemble bile-ducts. In toxic cirrhosis the connective tissue thickens from contraction, but does not increase in amount because the fibroblasts have not been injured. In infective cirrhosis the fibroblasts are destroyed along with the liver cells; hence there is much active regeneration and production of fibrous tissue. In syphilitic infection it is the fibroblasts which suffer most. When these rules are kept in mind, he believes it is possible to identify the type of cirrhosis microscopically.

### A New Drug for Infantile Diarrhœa.

In the *Archives of Pediatrics*, Dr. Vipond, of Montreal, advocates very strongly the merits of divi-divi as a drug for astringing the intestines in cases of "summer diarrhœa." This drug he now always orders in preference to bismuth or any other; and the preparation he prefers is the tincture, made by percolation and allowed to stand for five or six days before being filtered. This tincture of divi-divi contains a large percentage of tannin, as well as some unknown oleoresins which are believed to take a share in the astringent action produced. The author begins with half a drachm of the tincture in a little water every two hours, increasing to a drachm if the patient is over two months of age. The medicine has a distinctly astringent but not very unpleasant taste; to prevent refusal or vomiting it is given slowly, a few drops at a time, with a sip of boiled water between each portion of the dose to keep the astringent taste off the palate. Before starting this treatment a preliminary dose of castor oil is useful. The drug may produce an immediate effect; in many cases diarrhœa ceases after one dose, though sometimes the treatment has to be persevered with for some time. As for diet, the author is giving up the barley water and egg albumen *régime* in favour of a gruel made from banana flour. The tincture of divi-divi has also been used for adults with equal success. Divi-divi, it may be mentioned, is a tree which grows wild in the West Indies and in tropical America, and it is from the fruit or pods that the tincture is manufactured.

### Posture in Obstetric Cases.

In a lecture by Dr. Brodhead on the physician's duty in obstetric cases, published in the International *Journal of Surgery*, the author mentions with approval the suggestions of King as to the effects of posture in correcting malpresentations. It is held, for example, that a transverse lie can generally be corrected by getting the patient into a squatting posture, the anterior foot corresponding to the side to which the breech is directed; in other words, if the breech is to the left, the left foot should be forward. If the patient will assume this posture for some minutes, long enough for the uterus to contract several times, King believes that the thigh pressure will cause a change of presentation to the vertex. Again, if the case be one of persistent occipito-posterior, he advises kneeling in such a way that the pelvis rests upon the heels. It is claimed that the pressure of the heels on a point over the great sacrosciatic foramen will cause a forward rotation of the occiput. Before using forceps, the same author advises placing the patient in the exaggerated lithotomy position, with the thighs held up firmly against the abdominal walls; for this increased pressure will sometimes result in advance of the head. The usefulness of the knee-chest and lateral-prone postures in the treatment of prolapsed cord is too familiar to need emphasis. Another noteworthy point in Dr. Brodhead's lecture is his practice of giving a dose of ergot by the mouth immediately after the end of the second stage of labour.

### An Unusual Case of Late Pregnancy.

In the *Journal of the American Medical Association* Dr. C. C. Norris reports a case which shows in what unusual and unexpected patients pregnancy may occasionally occur. A woman of fifty had been twice married without becoming pregnant. After each marriage she had taken professional advice about her sterility, and on each occasion she had undergone simple dilatation and also dilatation with curetting without improving her powers of conception. Menstruation was always normal, and no especial dymenorrhœa or other symptom was complained of. The only abnormality discovered on examination was a small external os. Notwithstanding the age of the patient, the author decided once more to perform a dilatation, since the menopause had not yet taken place. After dilating the cervix ten days before an expected period, he left in a grooved-stem pessary known as the Wiley drain. These measures were followed by conception, and the pregnancy proceeded naturally to a normal labour and delivery.

### Thiosinamine.

The value of thiosinamine (fibro-lysin) as a solvent of cicatricial tissue is much called into question, so that Dr. Louis Rénon's recent communication to the Paris Academy of Medicine on his personal experiences with the drug in two hundred cases is of interest. He finds that it is soluble in water to 5 to 6 per cent. The solvent action on experimental cicatrices in rabbits, guinea-pigs, and dogs is very doubtful. In cerebro-medullary sclerosing affections and in the course of spasmodic paraplegias it occasionally determines a diminution in the contractures and brings relief from pain in cases of tabes. Sometimes it arrests the progressive evolution of chronic rheumatism. In pulmonary emphysema and in pulmonary and pleural sclerosis it diminishes dyspnœa to a noticeable extent. In cardio-vascular affections its action is very variable. No effect is obtained with it in mitral disease. In chronic aortitis, in aortic narrowing and insufficiency, no modification in the stethoscopic signs is to be noted after its exhibition, but dyspnœa is often relieved. The same is true of cardiac stenosis with or without mediastinitis. In arterio-sclerosis, headache and dyspnœa are often diminished, but arterial tension is reduced only after prolonged use of the drug, and then but slowly. It is contraindicated in tuberculous patients. It causes no unpleasant symptoms when given either by the mouth or by injections in daily doses of 6, 8, and 10 centigrams. The author considers it a drug of secondary importance capable of rendering certain help in therapeutics, but without giving the brilliant results claimed for it by some writers.

### Exhaust Gases of Gas Engines.

Professor Delepine publishes in *Public Health* an article on the pathogenic properties of the gases discharged by the exhaust pipe of gas engines. As the result of experiments, he comes to the following conclusions: The main cause of danger when the engine is overloaded is the considerable reduction in the amount of oxygen contained in the exhaust gas; the proportion of carbon dioxide is also very large. The main cause of danger when the supply of air to the experimental gas engine is insufficient is the presence of carbon monoxide. This begins to be manifest when the proportion of exhaust gas reaches 1 part of gas to 40 or 50 parts of air. It is probable, therefore, that the use of power gas is attended with greater danger than the use of ordinary lighting gas. Anything blocking the entrance of the air-pipe is sufficient to bring this about. It is obvious from this that care should always be taken that the exhause pipe of a gas engine does not directly or indirectly discharge its contents into more or less confined spaces to which persons may have access. The discharge of exhaust gases into sewers should be entirely forbidden.

